# Construction of a Fish‐like Robot Based on High Performance Graphene/PVDF Bimorph Actuation Materials

**DOI:** 10.1002/advs.201500438

**Published:** 2016-03-31

**Authors:** Peishuang Xiao, Ningbo Yi, Tengfei Zhang, Yi Huang, Huicong Chang, Yang Yang, Ying Zhou, Yongsheng Chen

**Affiliations:** ^1^ Centre for Nanoscale Science and TechnologyKey Laboratory of Functional Polymer MaterialsCollaborative Innovation Center of Chemical Science and Engineering (Tianjin)School of Materials Science and EngineeringNankai UniversityTianjin300071P.R. China

**Keywords:** actuator, fish, graphene, poly‐(vinylidene fluoride), robot

## Abstract

Smart actuators have many potential applications in various areas, so the development of novel actuation materials, with facile fabricating methods and excellent performances, are still urgent needs. In this work, a novel electromechanical bimorph actuator constituted by a graphene layer and a PVDF layer, is fabricated through a simple yet versatile solution approach. The bimorph actuator can deflect toward the graphene side under electrical stimulus, due to the differences in coefficient of thermal expansion between the two layers and the converse piezoelectric effect and electrostrictive property of the PVDF layer. Under low voltage stimulus, the actuator (length: 20 mm, width: 3 mm) can generate large actuation motion with a maximum deflection of about 14.0 mm within 0.262 s and produce high actuation stress (more than 312.7 MPa/g). The bimorph actuator also can display reversible swing behavior with long cycle life under high frequencies. on this basis, a fish‐like robot that can swim at the speed of 5.02 mm/s is designed and demonstrated. The designed graphene‐PVDF bimorph actuator exhibits the overall novel performance compared with many other electromechanical avtuators, and may contribute to the practical actuation applications of graphene‐based materials at a macro scale.

## 1 Introduction

Smart stimulus‐responsive materials can spontaneously respond to external stimuli, such as heat,^1^ electric,^2^ light,^3^ and other external stimuli,^4^, ^5^ and it also can convert these different energy forms to mechanical energy.^6^, ^7^ Especially, electromechanical actuators that can directly convert electrical energy to mechanical energy through mechanical response of materials, have been intensively developed and utilized over the past decades due to the fascinating properties and various applications, such as switches, sensors, artificial muscle, microrobotics, and other nano/micro electromechanical systems.^8^, ^9^, ^10^, ^11^, ^12^, ^13^, ^14^, ^15^, ^16^ In the recent years, electroactive polymers (EAPs) including dielectric elastomers,^17^ piezoelectric polymers,^18^ electrostrictive materials,^19^ ionic polymer metal composites,^20^ and conducting polymers,^21^ have been widely investigated and utilized as actuator materials due to the merits of low weight, high flexibility, and large deformation.

Graphene has been a promising material for various device applications since its discovery because of its superb electronic, thermal/chemical stability, and mechanical properties.^22^, ^23^, ^24^, ^25^, ^26^, ^27^ In recent years, carbon nanomaterial‐based multilayer polymeric actuators stimulated by light, electrochemical, and electric, etc., have attracted wide attention. For instance, Jiang et al. fabricated a polymeric bimorph biomimetic platform which could be reversibly deflected at millimeter scale within 3.4 s in response to near infrared irradiation.^3^ Kotal et al. report a highly efficient metal‐free sulfur and nitrogen codoped graphene electrode for economically viable, largely bendable, air‐working, and highly durable ionic polymer actuators.^15^ Recently, Hu et al. demonstrated an electromechanical bimorph actuator, which could exhibit the ability of undergoing large deformation under a low voltage (10 V) in 3 s.^28^ Though these carbon nanomaterial‐based multilayer polymeric actuators may have some potential applications, the overall excellent performances with short response time, large deformation, high generated stress, low‐driven voltage, and more universal operation environment are still challenges.

Herein, we demonstrate a novel electromechanical actuator which is composed of one layer of poly‐(vinylidene fluoride) (PVDF) and the other layer of flexible graphene paper, utilizing a facile process which only involves sequential filtration processing for graphene layer and drop‐coating processing for PVDF layer, respectively. PVDF is chosen as the polymer layer for several reasons: (i) PVDF as an electroactive polymer possesses excellent electrostrictive performance,^29^, ^30^, ^31^ (ii) PVDF as a representative semi‐crystalline polymer, the β phase exhibits a polarization vector different from zero, which makes PVDF have good converse piezoelectric effect performance,^19^, ^32^, ^33^, ^34^ (iii) Moreover, compared to other EAPs, PVDF has a large thermal expansion coefficient.^35^, ^36^ These properties make PVDF a popular material for EAP actuators which can rapidly response to high frequencies, but make smaller deflection or smaller strain at a high voltage.^19^, ^33^, ^34^, ^37^ Excitingly, the graphene–PVDF bimorph actuator that we demonstrate here can be reversibly deflected at a large scale in a few hundred milli­seconds upon electrical stimulus. When the sample (length: 20 mm, width: 3 mm) is applied to the voltage of 13.0 V, the tip displacement can be up to 14.0 mm in 0.262 s. And the specific stress generated from the deflection can be up to 312.7 MPa g^−1^ at the voltage of 17.0 V. The excellent performance of the bimorph actuator may provide a possible design for the construction of the robot. Subsequently, a fish‐like robot inspired by the dolphin swimming is designed, which is driven by the bimorph actuator upon electrical stimulus, and the fish‐like robot can swim at different frequencies, whose swimming speed is up to 5.02 mm s^−1^ when the voltage is 13.0 V.

## 2 Results and Discussion

### 2.1 Design and Fabrication of the Graphene‐PVDF Bimorph Actuator

The graphene film was first fabricated by direct filtration of an aqueous suspension of reduced graphene oxide colloids followed by 400 °C annealing according to previous works.^38^, ^39^ As already investigated, this graphene paper features with excellent flexibility, high electric, and thermal conductivity.^8^, ^38^ The graphene has a negative coefficient of thermal expansion, about −7.0 × 10^−6^ K^−1^,^25^, ^40^, ^41^ compared with the much larger thermal expansion coefficient of PVDF at about 130 × 10^−6^ K^−1^.^35^, ^36^ This may result in a deflection. What's more, PVDF has excellent converse piezoelectric effect and electrostrictive property, which can enhance the actuation performance at a large scale. When stimulated by the voltage, the bimorph film will deflect to the graphene side.

**Figure 1:**
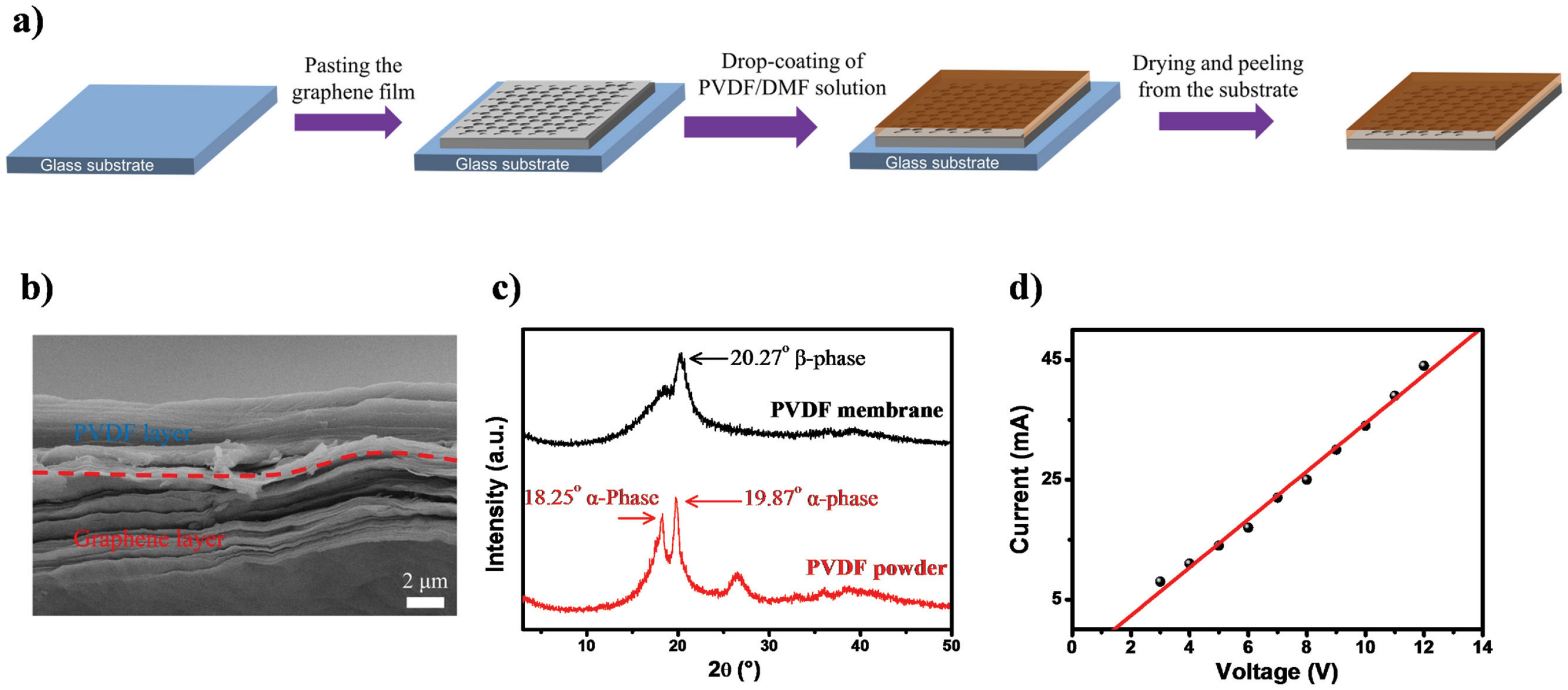
a) Schematic illustration of synthesis process of the graphene‐PVDF bimorph actuator. b) The cross‐sectional SEM images of the bimorph actuator. c) The XRD patterns of the PVDF powder and the PVDF membrane stripped from the graphene‐PVDF bimorph actuator. d) The DC current–voltage (*I*–*V*) curve of the graphene‐PVDF bimorph actuator, the correlation factor *R* of the linear line is 0.991.

**Figure** **1**a schematically illustrates the fabrication process of graphene‐PVDF bimorph actuator. The graphene film was first anchored onto a precleaned glass substrate. Subsequently, the PVDF/DMF (dimethylformamide) solution was drop‐coated onto the graphene film. The graphene‐PVDF bimorph actuator was obtained after the drying process and the peeling process from the glass substrate. Note that a very small amount of polyvinyl pyrrolidone (PVP) was added into the PVDF/DMF solution to enhance the adhesion force between the PVDF layer and the graphene layer. The strong specific dipolar interaction between the PVP's carbonyl group and the PVDF's fluorine group makes PVDF high compatible with reduced graphene oxide.^42^ The details about the fabrication process are described in the Experimental Section.

### 2.2 Characterization of the Graphene‐PVDF Bimorph Actuator

Figure 1b shows the representative side‐view SEM (Scanning electron microscope) image of the cross‐section of the graphene‐PVDF bimorph actuator. And the bimorph structure was seen clearly, consisting of one graphene layer with well‐packed layered structure and the other layer of PVDF. The thickness of the graphene layer and the PVDF layer is about 4–5 μm and 3–4 μm, respectively. The XRD (X‐ray diffraction) patterns of the PVDF powder and the PVDF membrane stripped from the graphene‐PVDF bimorph actuator are shown in Figure 1c, and the peak in 20.3° indicates the formation of the PVDF β phase, which exhibits good converse piezoelectric properties.^32^ In addition, FTIR (Fourier Transform infrared spectroscopy) measurement shows the typical peaks (840 cm^−1^) of the β phase, as shown in Figure S1 (Supporting Information). Moreover, the graphene–PVDF bimorph actuator exhibits a good conductivity of ≈2500 S m^−1^. Figure 1d demonstrates the current–voltage (*I*–*V*) curve of the graphene‐PVDF actuator. The linear line indicates that the resistance is relatively stable in the test range from 3 to 13 V. The square resistance of the graphene layer is also measured, about 58.79 Ω □^−1^, and this can be well in consistent with the data in the Figure 1d. In addition, the Raman spectra of the graphene layer on the different sites is shown in the Figure S6 a,b (Supporting Information), and it demonstrates that the graphene layer is very uniform.

### 2.3 Electromechanical Actuation of the Graphene‐PVDF Bimorph Actuator under Direct Current (DC)

**Figure 2:**
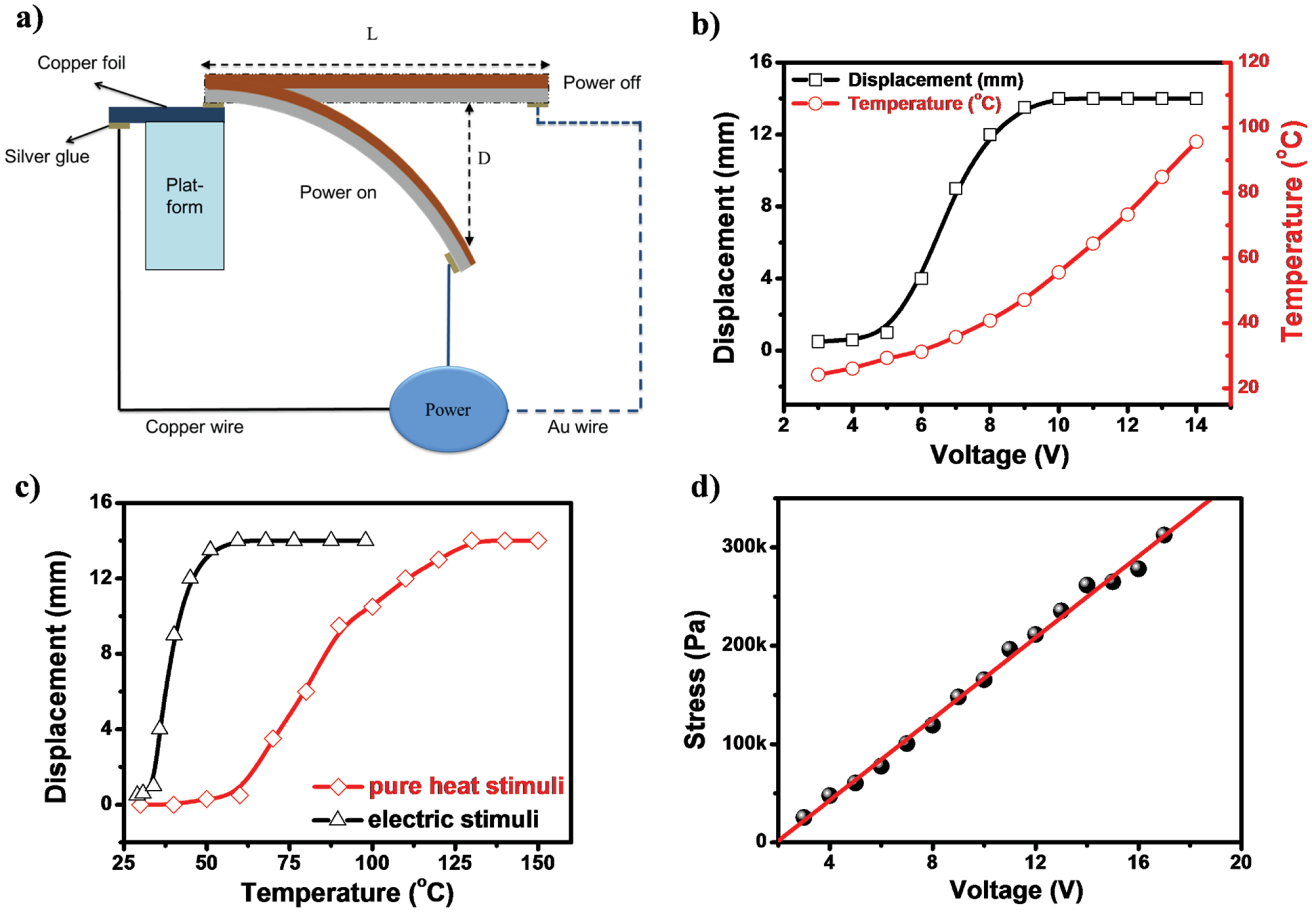
a) The experimental setup: when the power is on, the bimorph actuator will bend down; when the power is off, the actuator will bend back to its initial position. b) The displacement and temperature variation of the graphene‐PVDF bimorph actuator with changing the applied voltage. c) The tip displacement variations with the sample temperature under electric stimulus (△) and pure heat stimulus (◊). The dimensions of all the graphene‐PVDF bimorph actuator are 20 × 3.0 mm^2^ (length × width). d) The stress variation under different voltages using DC.

To investigate the actuation performance of the graphene‐PVDF bimorph actuator, a homemade setup was designed and demonstrated, as shown in **Figure** **2**a. One end of the graphene‐PVDF bimorph actuator was fixed to the Cu foil; the other end of the actuator was joined with a flexible Au wire. Accordingly, when the graphene‐PVDF bimorph actuator was applied the voltage, the end joined with the flexible Au wire would move freely and the actuator would bend to the side of the graphene layer because of the large expansion of the PVDF layer and the small shrinkage of the graphene layer.

In order to investigate the electromechanical properties of our graphene‐PVDF bimorph actuator, the thickness and the size of the actuator were all varied and optimized. First, pieces of the bimorph actuator with the thicknesses of graphene and PVDF layers being 4–5 μm and 3–4 μm, respectively, were cut into different sizes. In Figure S2 (Supporting Information), the *D*/*L* (displacement/length) ratio of samples with different sizes was similar. Therefore, the length–width ratio has no influence on the deflection angle of the graphene‐PVDF bimorph actuator. Subsequently, the samples with different thicknesses of PVDF layer were prepared while the sizes of the samples are uniform and the thickness of the graphene layer is 4–5 μm. The results are shown in Figure S3 (Supporting Information). The tip displacement *D* gets its maximum value when the thickness of the PVDF layer is 3–4 μm. So the samples in the following tests are all 4–5 μm for the graphene layer, and 3–4 μm for the PVDF layer.

When applied the direct current, as shown in Figure 2b, the tip displacement and the temperature of graphene‐PVDF bimorph actuator as functions of electric voltage variations were first investigated. And an infrared camera was employed to capture the temperature distribution of the sample, and then read the real‐time temperature in the center of the samples upon direct current, as shown in the Figure S7a,b (Supporting Information). With the increase of the voltage, the tip displacement was gradually enhanced and then got to a steady value of 14.0 mm. And it does not increase with the increase of the voltage or temperature any more. To note that if the voltage or temperature was further increased, the sample would be damaged, such as layered or burnt because of the high temperature produced by the graphene layer. According to our most of results, when the voltage exceeds 17 V, the paper will be damaged. The sample temperature, however, increased in a quadratic form because of the Joule heat, *P* =*U* ^2^/*R*. Thus, the tip displacement variation has a mismatch with the variation of the sample temperature. Accordingly, it might be possible that the temperature change induced by the Joule effect was one of the motivations to the deflection due to the different coefficients of thermal expansion (CTE) of the two layers.

To further investigate the influence of the temperature change on the deflection, we extracted the temperature data from the infrared camera, and made the tip displacement of the cantilever beam as a function of the temperature. As a contrast, we also carried out a study on the deflection in which the graphene‐PVDF bimorph actuator was heated in an oven. As shown in Figure 2c, under the electrical stimulus, the tip displacement increased very quickly accompanying an increase of the sample temperature as expected, then it got to the maximum value of 14.0 mm at 55 °C, and kept at steady even with further increasing the voltage. While under the control experiment with only heat stimulus, the tip displacement was gradually increased and then got to the maximum, 14.0 mm, but at a much higher temperature of 130 °C. It is important to note that, at the temperature of 55 °C the max displacement of 14.0 mm was achieved under the electrical stimulus, but the tip displacement under pure heat stimulus was only 0.5 mm. Thus, the deflection ratio induced by heat is only 3.6%. So, in addition to the thermal‐induced mechanism due to the different thermal expansion coefficient of the two layers, it is very likely that there is another mechanism for the enhanced displacement under the electrical stimulus. Based on our results, this mechanism could include the converse piezoelectric effect and electrostrictive performance of PVDF. First, under the external electric field, the dipoles induced in the dielectrics will align with the field, and the strain is induced by the polarization process. This phenomenon is the electrostrictive effect of PVDF which exists in all of the dielectrics, and the strain orientation is not relevant with the direction of the external electric field.^29^, ^30^, ^31^ Moreover, the PVDF β phase exhibits good converse piezoelectric properties under the external electric field.^19^, ^32^, ^33^, ^34^ So, under the external electric field, the total electrical‐induced strain of the piezoelectric materials is the result of the two strains induced by the electrostrictive effect and the converse piezoelectric effect, respectively. These effects largely should enhance the tip displacement. The mechanism also will be further discussed below.

To further evaluate the actuation performance of the graphene‐PVDF bimorph actuator, the electric‐induced stress of the actuator was investigated, and the results were depicted in Figure 2d. A home‐made experimental setup was designed according to our previous work to quantitatively measure the induced stress,^43^ as shown in Figure S5 (Supporting Information). Before carrying out the actuation stress test for all actuator samples, the fine‐tuning device was tuned to preload a small force (0.0196 N) on the graphene‐PVDF bimorph actuator to make sure that the samples were vertical to the horizontal plane of the balance. As indicated in Figure 2d, the generated stress increased with the increasing of the voltage. The generated maximum stress was up to about 312.7 KPa with a voltage of only 17.0 V. This is approximate to the peak capacity of the natural muscle (≈0.4 MPa).^8^ Remarkably, the generated specific stress (normalized by the mass of the total actuator samples) for the graphene–PVDF bimorph actuator can reach 312.7 MPa g^−1^ under 17.0 V, which is larger than the tensile strength of traditional engineering polymer materials.

### 2.4 Electromechanical Actuation of the Graphene‐PVDF Bimorph Actuator under Alternating Current (AC)

**Figure 3:**
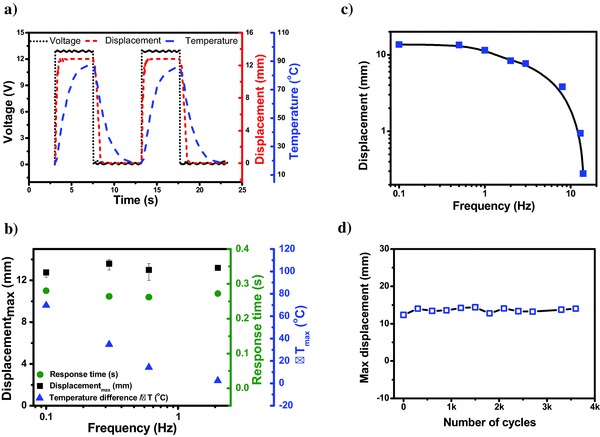
a) The real‐time displacement and temperature versus the time for two cycles under square wave AC input with the voltage of 0–13.0 V and frequency of 0.1 Hz. b) The max tip displacement (▪), the response time (●), and the temperature difference (▴) comparisons under different frequencies (0.1, 0.3, 0.6, and 2.0 Hz) with the same voltage input. c) The tip displacement as a function of the frequency of the applied square wave voltage (0–13.0 V). d) The cycle life testing at a 0.5 Hz square wave voltage (0–13.0 V). The dimensions of all the graphene‐PVDF bimorph actuator are 20 × 3.0 mm^2^ (length × width).

The real‐time displacement and temperature variation of the graphene‐PVDF bimorph actuator with the cycled voltage on and off were simultaneously monitored. When applied the square wave voltage (0–13.0 V), the graphene‐PVDF bimorph actuators could be triggered, and exhibited rapid and reversible swing motions, also shown in Video S1 (Supporting Information). The tip displacement variation and the temperature variation under different frequencies (0.1, 0.3, 0.6, and 2.0 Hz) were studied. **Figure** **3**a shows the variation under the frequency of 0.1 Hz, and the results under other frequencies were shown in Figure S4 (Supporting Information). When the voltage varied from 0–13.0 V, the displacement took only about 0.280 s to reach the maximum value (under 0.1 Hz). The response time of the graphene‐PVDF bimorph actuator is much faster than many other graphene‐based bimorph electromechanical actuators.^3^, ^28^, ^44^ However, the temperature was gradually increased to the maximum 82 °C, and the max temperature difference is 64.1 °C (the initial temperature is 17.9 °C). As a contrast, shown in Figure 3b, under the four frequencies the vibration is quite reversible with nearly the same amplitude displacement, and the response time is almost consistent too, but the temperature difference decreased with the frequency increasing, as shown in Table S1 (Supporting Information). Especially, under the frequency of 2.0 Hz, when the power was off, the tip displacement was 0 mm, and the temperature was 47.1 °C. When the power was on, the tip displacement reached the maximum about 13.2 mm rapidly and the temperature only increased by 2.2 to 49.3 °C. Thus, as speculated above (Figure 2c), this pheno­menon also indicates that the actuation performance was not just induced by the electric–thermal conversion, but also by the converse piezoelectric effect and electrostrictive performance of PVDF. This also can be further supported by the calculation section using the model demonstrated in the Figure S8 (Supporting Information).

The frequency response and the cycle life are two significant indexes for evaluating the performance of actuators. Figure 3c shows the vibration amplitude of the graphene‐PVDF bimorph actuator at the square wave voltage (0–13.0 V) with different frequencies. Under the frequency of 0.1 Hz and the voltage of 0–13.0 V, the bimorph actuator generated the displacement amplitude of 14.0 mm, similar as that in DC test. With the frequency increasing, the vibration amplitude gradually reduced. Moreover, the vibration with the frequency up to 14 Hz could still be observed. Furthermore, the reliability and repeatability of the swing actuation performance for the actuator were also investigated. As demonstrated in Figure 3d, the graphene‐PVDF bimorph actuator was tested for more than 3600 swing cycles with the frequency of 0.5 Hz and the square wave voltage of 0–13.0 V continually, and no downgraded actuation performance was observed. These results indicate that our graphene‐PVDF bimorph actuators are quite reversible and stable, and have many potential applications.

### 2.5 Construction of the Electric‐Driven Fish‐Like Robot

**Figure 4:**
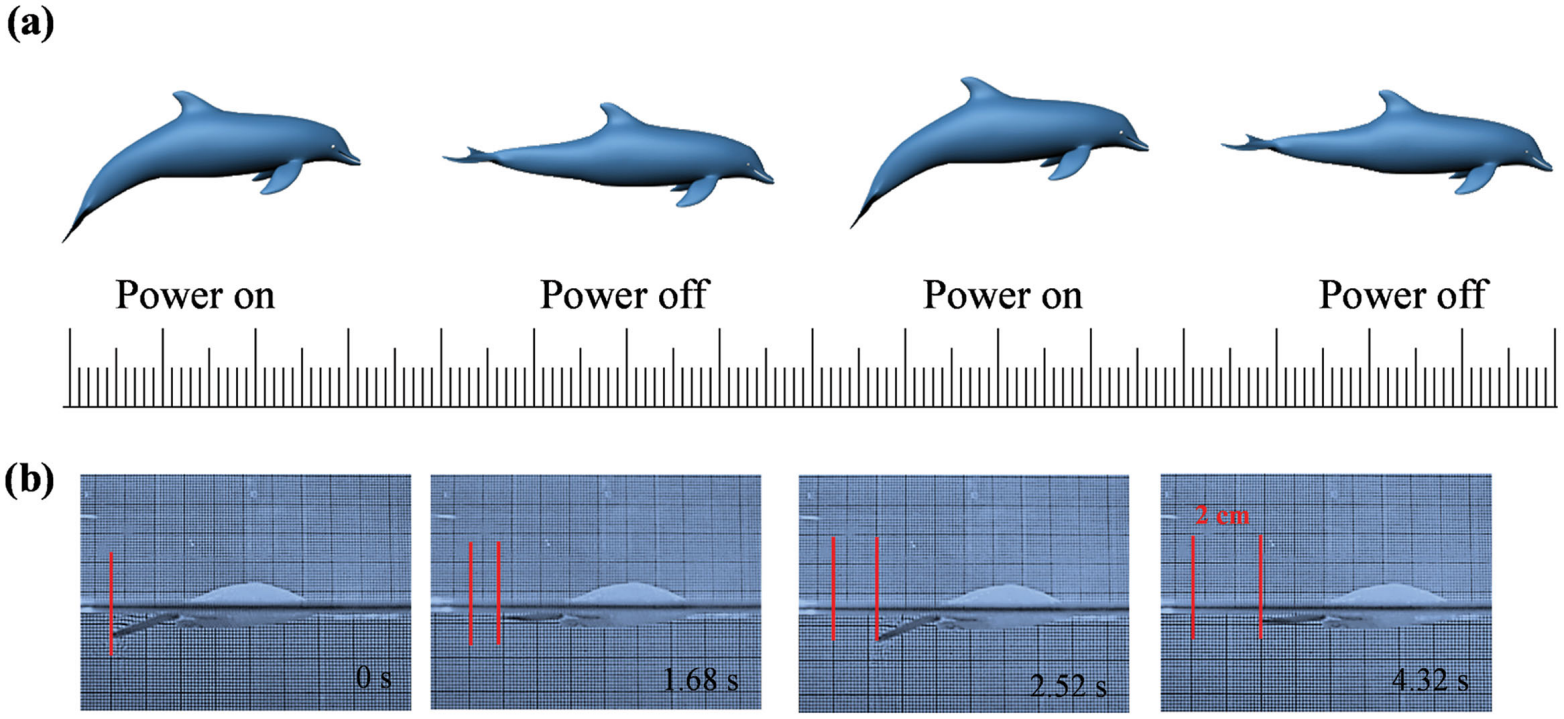
a) Diagram to demonstrate the fish‐like robot swimming, when the power is on or off, the “tail” bends down or up, then the fish‐like robot will swim forward. b) The optical images of an artificial fish‐like robot. The dimensions of the fish tail (the graphene‐PVDF bimorph robot and the fish body (Expandable polystyrene) are 14 × 3 mm^2^ and 30 × 8 mm^2^, respectively. The fish‐like robot moves from left to right at a speed of 5.02 mm s^−1^ applied the voltage of 0–13.0 V and the frequency of 0.4 Hz.

The graphene‐PVDF bimorph actuator with the ultra large displacement and fast response to the low electric voltage could provide a basis for the various robotic device applications. Herein, a fish‐like robot was designed to imitate fish swimming in petroleum ether. The fish‐like robot consists of an expandable polystyrene body and a tail which is made of the graphene‐PVDF actuator to offer the propulsion. As shown in **Figure** **4**a, both of the ends of the graphene‐PVDF film are connected with a flexible Au wire as the conductive wire. When the power was on, the tail would bend down. When the power was off, the tail would bend up to the initial position. Thereby, the fish‐like robot would swim forward like a real dolphin. The moving speed could be calculated, up to 5.02 mm s^−1^, shown in Figure 4b. It is important to note that, due to the lightness and chemical/thermal stability, the robot based on this design could be used in harsh chemical environments.

## 3 Conclusions

In summary, the electric‐driven bimorph actuator comprised of one layer of graphene and another layer of PVDF was fabricated. The actuator could bend to the graphene layer side with a maximum deflection of about 14.0 mm within 0.262 s, it also could generate large stress (>312.7 MPa g^−1^). In addition to the conventional thermal‐induced mechanism (the different CTEs of two layers), other mechanisms including the good converse piezoelectric effect and electrostrictive performance of PVDF, also play a significant role in the fast and enhanced actuation. Interestingly, the actuator could exhibit fast and reversible swing motions without any downgrade performance due to the super mechanical properties of graphene. The actuation could response up to 14 Hz while the performance keeps unchanged with even high cycling times (over 3600 times). Finally, based on the graphene‐PVDF bimorph actuator, a fish‐like robot was built, and it could swim at the speed of 5.02 mm s^−1^. Note that better performance is expected with further material and device structure optimization. These results may have some important implications to design micro or light devices based on graphene for applications in various areas such as for sensors, microrobotics, switches, artificial muscle, and so on.

## Supporting information

As a service to our authors and readers, this journal provides supporting information supplied by the authors. Such materials are peer reviewed and may be re‐organized for online delivery, but are not copy‐edited or typeset. Technical support issues arising from supporting information (other than missing files) should be addressed to the authors.

Supplementary

Supplementary

Supplementary

## References

[1] S. E. Zhu , R. Shabani , J. Rho , Y. Kim , B. H. Hong , J. H. Ahn , H. J. Cho , Nano Lett. 2011, 11, 977. 21280657 10.1021/nl103618e

[2] H. Cheng , Y. Liang , F. Zhao , Y. Hu , Z. Dong , L. Jiang , L. Qu , Nanoscale 2014, 6, 11052. 25144446 10.1039/c4nr03409k

[3] W. Jiang , D. Niu , H. Liu , C. Wang , T. Zhao , L. Yin , Y. Shi , B. Chen , Y. Ding , B. Lu , Adv. Funct. Mater. 2014, 24, 7598.

[4] D. D. Han , Y. L. Zhang , H. B. Jiang , H. Xia , J. Feng , Q. D. Chen , H. L. Xu , H. B. Sun , Adv. Mater. 2015, 27, 332. 25327686 10.1002/adma.201403587

[5] J. Liang , Y. Huang , J. Oh , M. Kozlov , D. Sui , S. Fang , R. H. Baughman , Y. Ma , Y. Chen , Adv. Funct. Mater. 2011, 21, 3778.

[6] Y. Zhao , L. Song , Z. Zhang , L. Qu , Energy Environ. Sci. 2013, 6, 3520.

[7] Y. Huang , J. Liang , Y. Chen , J. Mater. Chem. 2012, 22, 3671.

[8] R. H. Baughman , C. X. Cui , A. A. Zakhidov , Z. Iqbal , J. N. Barisci , G. M. Spinks , G. G. Wallace , A. Mazzoldi , D. De Rossi , A. G. Rinzler , O. Jaschinski , S. Roth , M. Kertesz , Science 1999, 284, 1340. 10334985 10.1126/science.284.5418.1340

[9] R. Pelrine , R. Kornbluh , Q. Pei , J. Joseph , Science 2000, 287, 836. 10657293 10.1126/science.287.5454.836

[10] R. H. Baughman , Synth. Met. 1996, 78, 339.

[11] A. Lendlein , H. Y. Jiang , O. Junger , R. Langer , Nature 2005, 434, 879. 15829960 10.1038/nature03496

[12] H. Koerner , G. Price , N. A. Pearce , M. Alexander , R. A. Vaia , Nat. Mater. 2004, 3, 115. 14743213 10.1038/nmat1059

[13] S. V. Ahir , E. M. Terentjev , Nat. Mater. 2005, 4, 491. 15880115 10.1038/nmat1391

[14] Y. Yang , W. Zhan , R. Peng , C. He , X. Pang , D. Shi , T. Jiang , Z. Lin , Adv. Mater. 2015, 27, 6376. 26389820 10.1002/adma.201503680

[15] M. Kotal , J. Kim , K. J. Kim , I. K. Oh , Adv. Mater. 2015, DOI: 10.1002/adma.201505243.

[16] Y. Hu , G. Wu , T. Lan , J. Zhao , Y. Liu , W. Chen , Adv. Mater. 2015, 27, 7867. 26498737 10.1002/adma.201502777

[17] S. Michel , B. T. T. Chu , S. Grimm , F. A. Nuesch , A. Borgschulte , D. M. Opris , J. Mater. Chem. 2012, 22, 20736.

[18] S. H. Bae , O. Kahya , B. K. Sharma , J. Kwon , H. J. Cho , B. Özyilmaz , J. H. Ahn , ACS Nano 2013, 7, 3130. 23448089 10.1021/nn400848j

[19] R. Pérez , M. Král , H. Bleuler , Sensor. Actuat. A 2012, 183, 84.

[20] L. Lu , W. Chen , Adv. Mater. 2010, 22, 3745. 20512819 10.1002/adma.201001134

[21] E. Smela , Adv. Mater. 2003, 15, 481.

[22] K. S. Novoselov , A. K. Geim , S. V. Morozov , D. Jiang , Y. Zhang , S. V. Dubonos , I. V. Grigorieva , A. A. Firsov , Science 2004, 306, 666. 15499015 10.1126/science.1102896

[23] A. K. Geim , K. S. Novoselov , Nat. Mater. 2007, 6, 183. 17330084 10.1038/nmat1849

[24] C. Lee , X. Wei , J. W. Kysar , J. Hone , Science 2008, 321, 385. 18635798 10.1126/science.1157996

[25] W. Bao , F. Miao , Z. Chen , H. Zhang , W. Jang , C. Dames , C. N. Lau , Nat. Nanotechnol. 2009, 4, 562. 19734927 10.1038/nnano.2009.191

[26] L. Gong , I. A. Kinloch , R. J. Young , I. Riaz , R. Jalil , K. S. Novoselov , Adv. Mater. 2010, 22, 2694. 20473982 10.1002/adma.200904264

[27] X. Huang , X. Qi , F. Boey , H. Zhang , Chem. Soc. Rev. 2012, 41, 666. 21796314 10.1039/c1cs15078b

[28] Y. Hu , T. Lan , G. Wu , Z. Zhu , W. Chen , Nanoscale 2014, 6, 12703. 25220910 10.1039/c4nr02768j

[29] Q. M. Zhang , V. Bharti , X. Zhao , Science 1998, 280, 2101. 9641912 10.1126/science.280.5372.2101

[30] F. Carta , Y. J. Hsu , J. Sarik , I. Kymissis , Org. Electron. 2013, 14, 286.

[31] O. R. Hughes , J. Polym. Sci. B‐Polym. Phys. 2007, 45, 3207.

[32] P. Martins , A. C. Lopes , S. Lanceros‐Mendez , Prog. Polym. Sci. 2014, 39, 683.

[33] C. C. Ma , Y. H. Huang , S. Y. Pan , Sensors 2012, 12, 2088. 22438754 10.3390/s120202088 PMC3304156

[34] J. M. Park , G. Y. Gu , Z. J. Wang , D. J. Kwon , K. L. DeVries , Appl. Surf. Sci. 2013, 287, 75.

[35] V. Speranza , R. Pantani , G. B. Besana , G. Titomanlio , Polym. Eng. Sci. 2007, 47, 1788.

[36] K. P. Pramoda , A. Mohamed , I. Y. Phang , T. Liu , Polym. Int. 2005, 54, 226.

[37] K. Y. Shin , J. Y. Hong , J. Jang , Chem. Commun. 2011, 47, 8527. 10.1039/c1cc12913a 21717029

[38] J. Liang , Y. Xu , D. Sui , L. Zhang , Y. Huang , Y. Ma , F. Li , Y. Chen , J. Phys. Chem. C 2010, 114, 17465.

[39] H. Chen , M. B. Müller , K. J. Gilmore , G. G. Wallace , D. Li , Adv. Mater. 2008, 20, 3557.

[40] C. Grigoriadis , N. Haase , H. J. Butt , K. Mullen , G. Floudas , Adv. Mater. 2010, 22, 1403. 20437491 10.1002/adma.200903264

[41] C. Chen , S. Rosenblatt , K. I. Bolotin , W. Kalb , P. Kim , I. Kymissis , H. L. Stormer , T. F. Heinz , J. Hone , Nat. Nanotechnol. 2009, 4, 861. 19893525 10.1038/nnano.2009.267

[42] V. Panwar , K. Cha , J. O. Park , S. Park , Sensor. Actuat. B 2012, 161, 460.

[43] J. Liang , L. Huang , N. Li , Y. Huang , Y. Wu , S. Fang , J. Oh , M. Kozlov , Y. Ma , F. Li , R. Baughman , Y. Chen , ACS Nano 2012, 6, 4508. 22512356 10.1021/nn3006812

[44] H. Bi , K. Yin , X. Xie , Y. Zhou , S. Wan , F. Banhart , L. Sun , Nanoscale 2013, 5, 9123. 23907556 10.1039/c3nr01988h

