# Supplementary material for: Construction of a Fish‐like Robot Based on High Performance Graphene/PVDF Bimorph Actuation Materials
Source: Adv Sci (Weinh). 2016 Mar 31;3(6):1500438. doi: 10.1002/advs.201500438 (PMC5071709; doi:10.1002/advs.201500438)
Supplement: Supplementary file 1 — Supplementary [file ADVS-3-0m-s001.pdf]

Copyright WILEY-VCH Verlag GmbH & Co. KGaA, 69469 Weinheim, Germany, 2016.

ADVANCED SCIENCE
Open Access

Supporting Information

for *Adv. Sci.*, DOI: 10.1002/advs.201500438

Construction of a Fish-like Robot Based on High Performance Graphene/PVDF Bimorph Actuation Materials

Peishuang Xiao, Ningbo Yi, Tengfei Zhang, Yi Huang, Huicong Chang, Yang Yang, Ying Zhou, and Yongsheng Chen**

Copyright WILEY-VCH Verlag GmbH & Co. KGaA, 69469 Weinheim, Germany, 2015.

Supporting Information

The construction of a fishlike robot based on high performance graphene/PVDF bimorph actuation materials

Peishuang Xiao, Ningbo Yi, Tengfei Zhang, Yi Huang*, Huicong Chang, Yang Yang, Ying Zhou, Yongsheng Chen*

Preparation of Graphene Papers.

According to our and other group's previous reports,[1, 2] graphene paper was fabricated through the below procedure. First, 25 mg of graphene oxide (the average size of 1-2 μm) prepared by the modified Hummer's method,[3, 4] was homogeneously dispersed in 100 mL of distilled water by ultrasonication. Then, 50μL of hydrazine hydrate (80%) and 350μL of ammonia solution (25%) were added to the above homogeneous solution. Subsequently, the solution was preliminarily reduced under 80 ^{o}C for 1.5 h. Graphene paper (the thickness of about 10 μm) was then obtained by filtration *via* an mixed cellulose membrane filter (50 mm in diameter, 0.45μm pore size; Ameritech), followed by vacuum drying and immersed in acetone to dissolve the filter. Finally, the graphene paper was annealed at 400 ^{o}C in argon for 1 h for further reduction, and the thickness would decrease because of the loss of oxygen-containing functional groups and the stack of the graphene layer.

Fabrication of Graphene-PVDF Bimorph actuator.

The PVDF powder and the PVP powder at a weight ratio of 20:1 were dissolved in DMF at a PVDF concentration of 25 mg/ml. Then, anchoring the graphene paper cut with the desired dimensions onto a substrate (glass or quartz), and subsequently drop-coating the PVDF solution followed by drying at 85 ^{o}C for 1 h. Then the graphene-PVDF bimorph actuator was obtained. Remarkably, the PVDF β phase is favorable to be formed in this method.[5-8] The

thickness of the PVDF layer can be modulated through controlling the amount of the PVDF solution drop-coated on the graphene. Then the bimorph actuator was cut into desired dimensions to make further measurement.

Measurement of Bending Performance and the Electric-induced Stress for the Actuator.

An experimental actuator setup, as demonstrated in Figure 2a, was designed and used to measure the electric-induced bending performance of the actuators. One end of the length of the graphene-PVDF bimorph actuator was fixed to an electrode of Cu foil; the other end of the film was connected to a flexible conductive Au wire. When the graphene-PVDF actuator was exposed to direct current or alternating current, the end joined with the Au wire can move and bend freely. The electric-induced stress was tested *via* an experiment setup according to our previous work as demonstrated in Figure S5.

Characterization.

The cross-sectional images of SEM were obtained on a JEOL JSM-7500F scanning electron microscope using an accelerating voltage of 5 KV or 20 kV. X-ray diffraction (XRD) measurements were carried out using a Rigaku D/Max-2500 diffractometer with Cu Kα radiation. Fourier Transform infrared spectroscopy (FTIR) was carried out using Bio-rad FTS-6000 FT-IR spectrometer. An OMRON laser displacement sensor (ZX-LD100) was used to record the tip displacement of this bimorph actuator. A visual infrared thermometer FLUKE-VT04 was employed to measure the real-time temperature change of the bimorph actuator measured from the graphene surface of the bimorph paper. The direct current was applied using a direct current regulated power supply, and the altering current was applied using a signal generator, which can alter the frequency from 0.1 Hz to 3 MHz. Rigol DS1102E digital oscilloscope (1 GSa s^{-1}, 100 MHz) was utilized to show the altering current form, such as the square wave voltage.

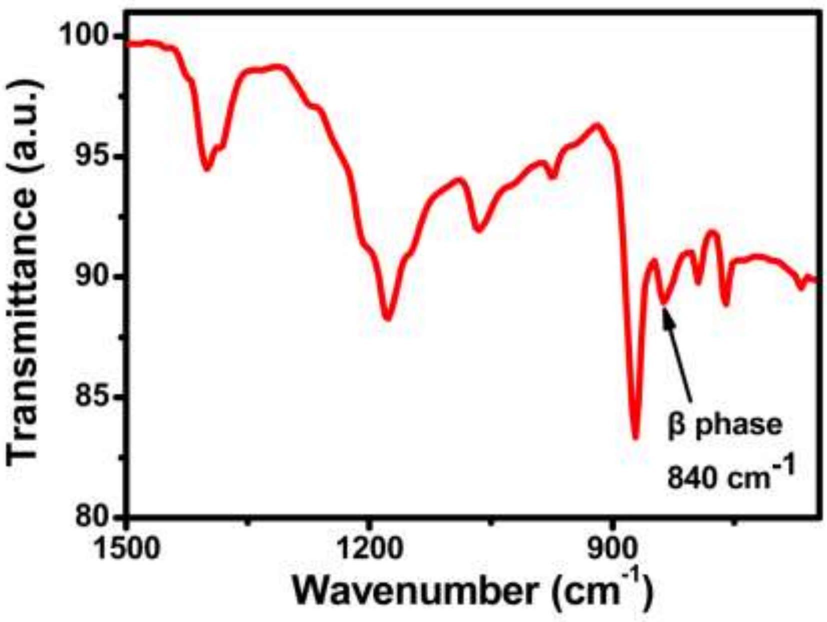

Figure S1. The FTIR spectra of the PVDF layer on the graphene-PVDF bimorph actuator.

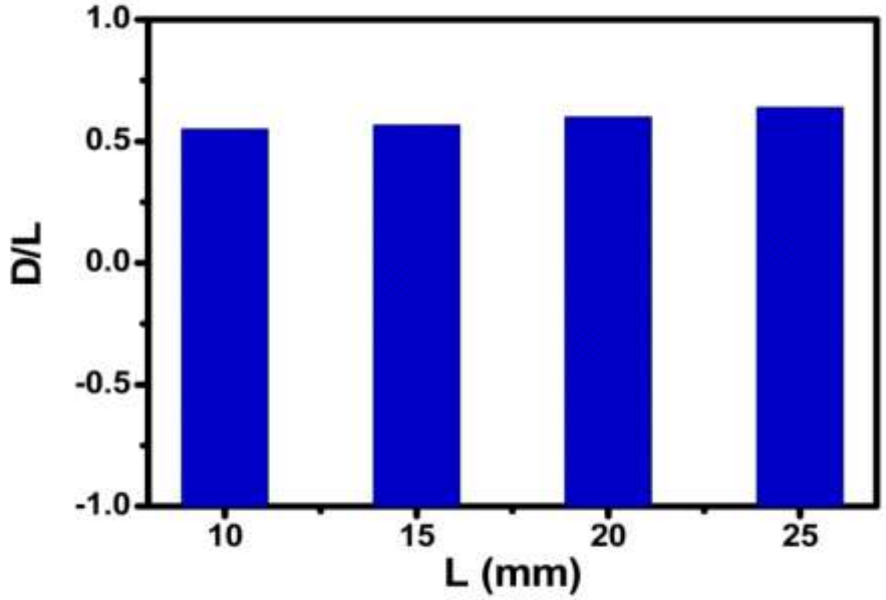

Figure S2. The ratio of displacement (D)/length (L) as a function of the sample length indicates that the deflection angle is no large variation with the increase of the length. The dimensions (length × width) of four different samples are 10 mm × 3 mm, 15 mm × 3 mm, 20 mm × 3 mm and 25 mm × 3 mm, respectively.

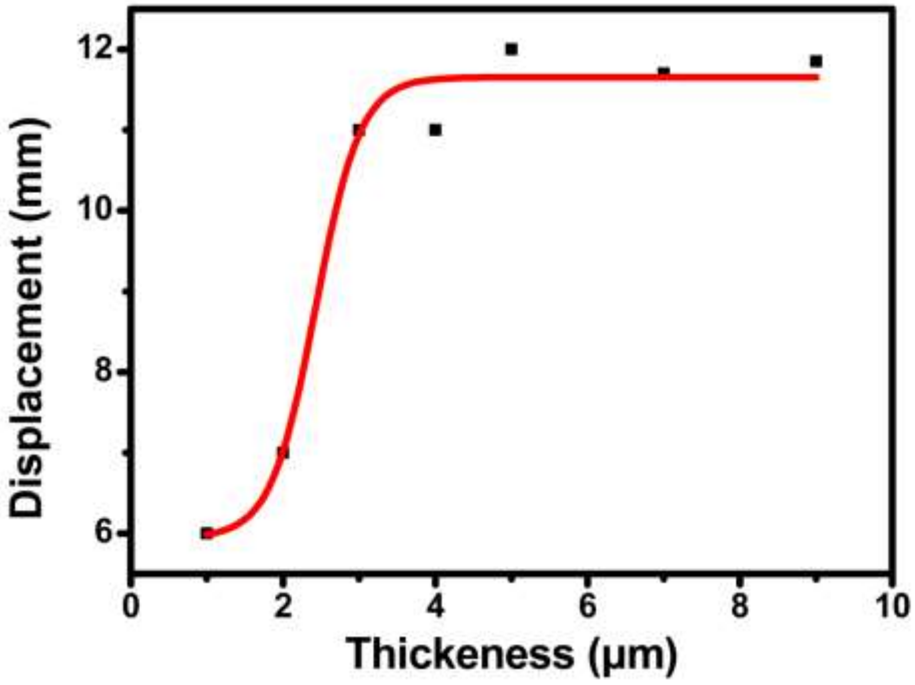

Figure S3. The thickness of the PVDF layer *vs* the max displacement of the graphene-PVDF bimorph actuator. The samples with the same dimensions: 18 mm × 3 mm.

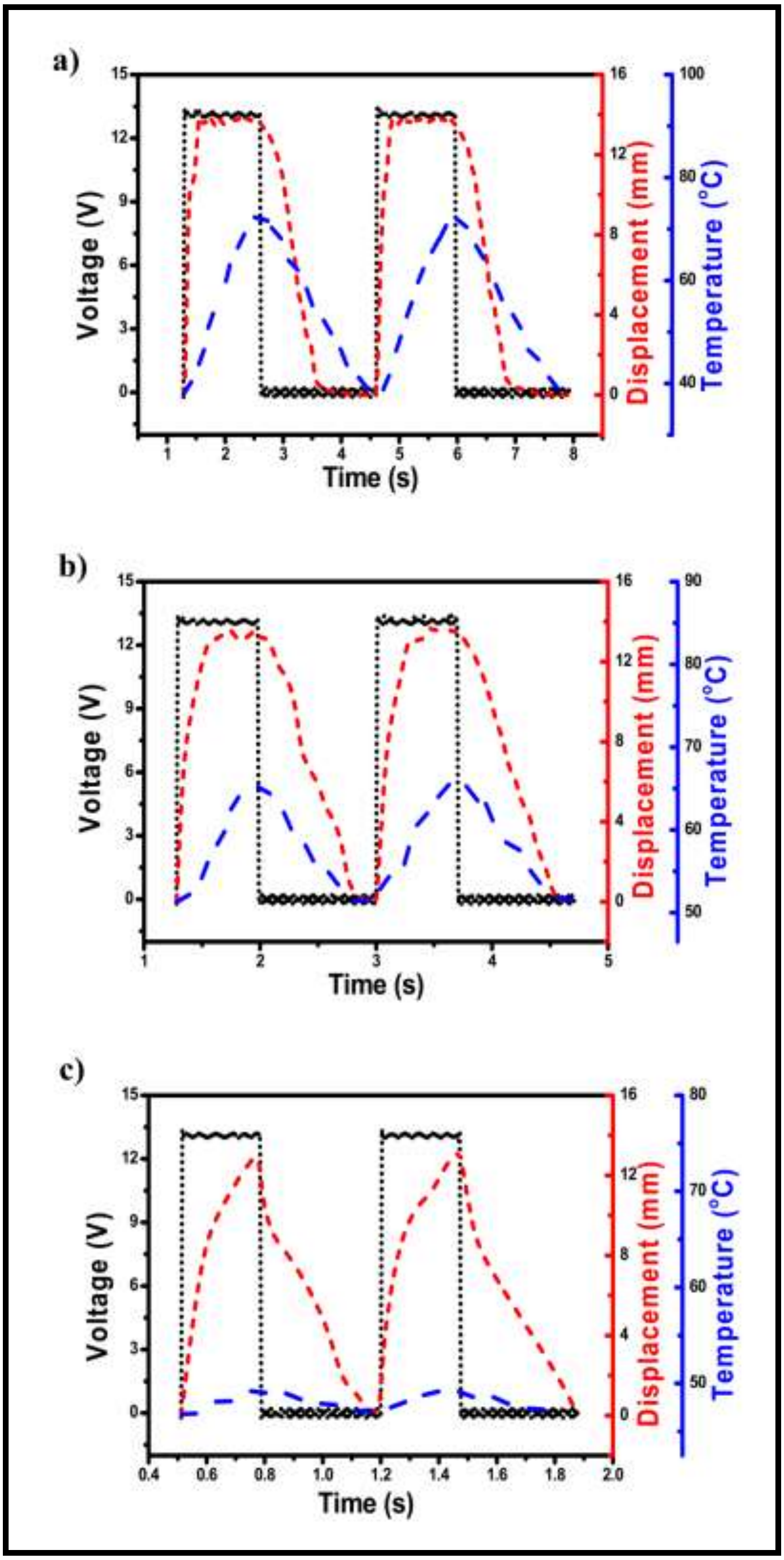

Figure S4. The real-time voltage, displacement, and temperature variations for two cycles under square wave input with the voltage of 0-13.0 V and excitation frequency of 0.3 Hz (a), 0.6 Hz (b), and 2.0 Hz (c).

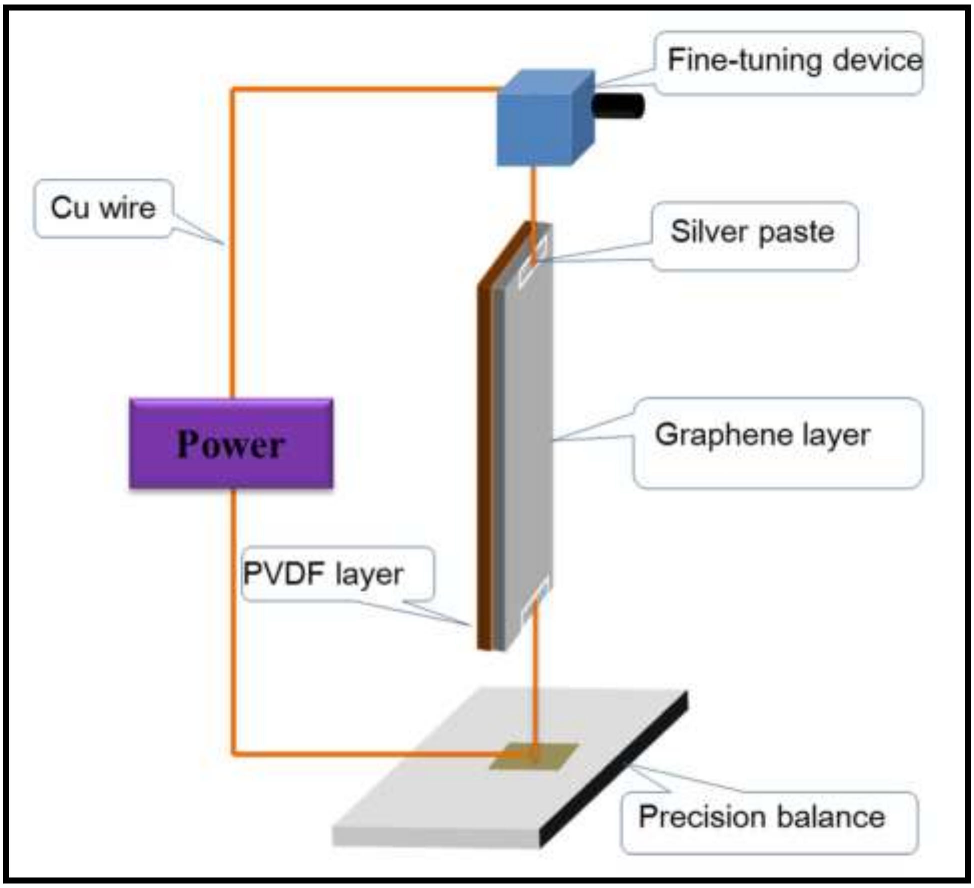

Figure S5. The experimental setup used for the measurement of the electric-induced stress of the Graphene-PVDF bimorph actuators

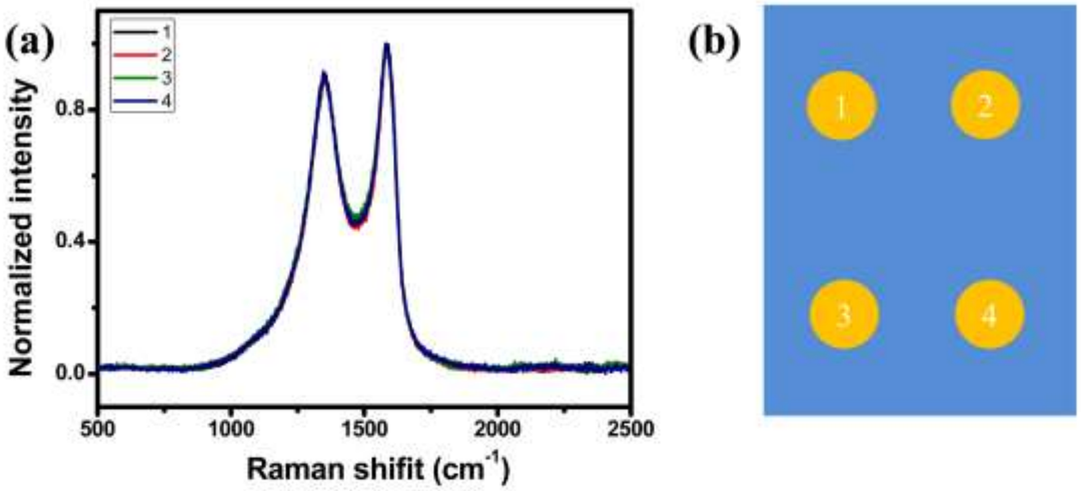

Figure S6. a) The Raman spectra of the graphene layer on the different sites in (b), and the I_D/I_G ratios are 0.895, 0.905, 0.908 and 0.907 from Sample 1 to Sample 4, respectively. b) the tested sites on the graphene layer of the sample(3.0 mm x 2.5 mm).

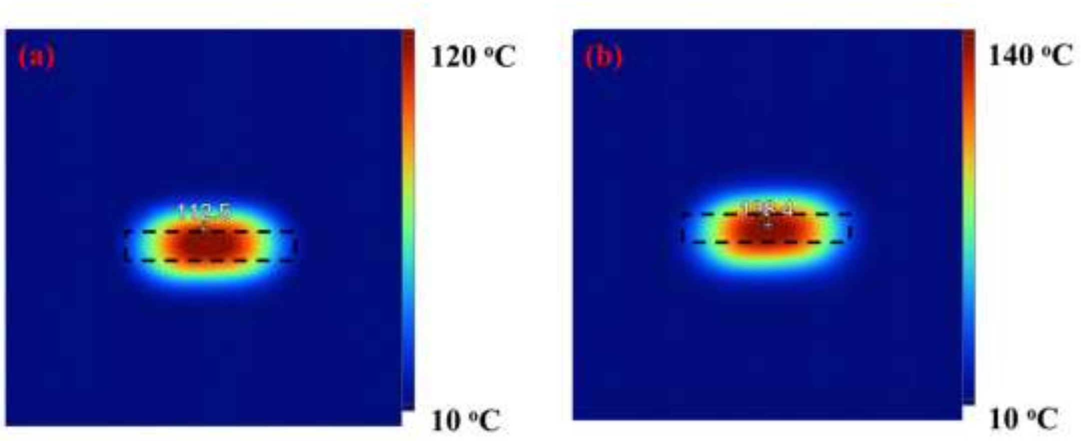

Figure S7. a) The temperature distribution of the sample (Length: 20 mm, Width: 3 mm) at 14 V; b) the temperature distribution of the sample (Length: 20 mm, Width: 3 mm) at 17 V. The dotted gray lines represent the bimorph actuator boundaries: inside is the actuator, outside is the background.

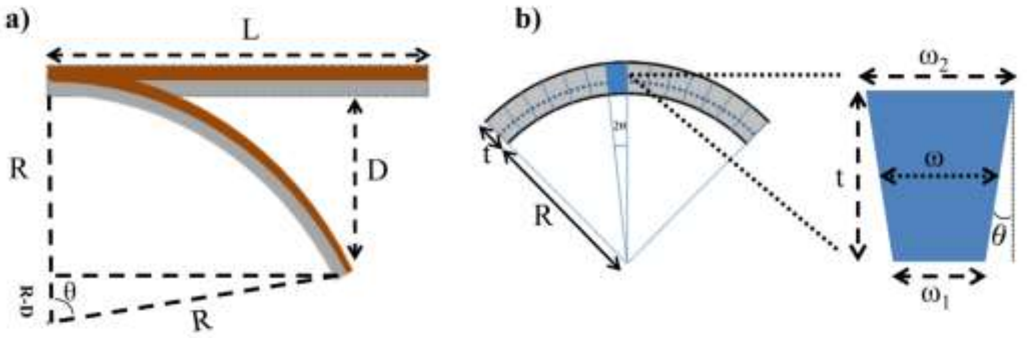

Figure S8. Two models for calculating the contribution amount of thermal expansion for the whole displacement.

According to the data from the results of 2.0 Hz, we can calculate the expansion amount induced by the thermal expansion. As shown in Figure S6, we utilized two models for our calculation and confirmation for the above mechanism. In the first model, the curvature is defined to be the reciprocal radius (1/R), as shown in Eq. (1).[9] In the second model, the curvature is shown in Eq. (2).[10]

$$k = \frac{2D}{I^2} \quad (1)$$

$$k = \frac{1}{t} \cdot \frac{w_2 - w_1}{w} \quad (2)$$

where the D, I, t, w_1, w_2 and w are the tip displacement, the length of the sample, the thickness of the sample, the PVDF layer length after deflection, the graphene layer length after deflection and the average length of the sample, respectively. Among those, w_1 is about equal to w because of the small shrinkage of the graphene layer.

According to model 1, the calculated curvature k is about 0.07 mm^{-1}. So in model 2, the calculated elongation w_2-w_1 is about 0.0112mm. The thermal expansion amount of the PVDF layer L_p is about 0.00562 mm, and the thermal shrinkage amount of the graphene layer L_g is about -3.08×10^{-4} mm, so the elongation induced by the thermal expansion L_p-L_g is about 0.00593 mm. Therefore, we can conclude that the contribution amount of the thermal-induced effect is about 52.9 %. Taking the calculation error into account, this result also supports that

the converse piezoelectric effect and the electrostrictive performance of PVDF contribute much to the deflection of the graphene-PVDF bimorph actuator.

Table S1. The comparison of the response time, the max displacement, the temperature difference and the max temperature difference values under four frequencies (0.1, 0.3, 0.6 and 2.0 Hz) applied the voltage of 0-13.0 V. The samples are all 20 mm × 3 mm (length × width). The temperature difference is defined as the corresponding value when the tip displacement reaches its maximum; the max temperature difference is defined as the corresponding value when the temperature reaches its maximum.

Frequency (Hz)	0.1 Hz	0.3 Hz	0.6 Hz	2.0 Hz
The response time (s)	0.28	0.264	0.262	0.272
The max displacement (mm)	12.7	13.6	13.0	13.2
The temperature difference (^{o}C)	5.9	4.4	5.4	2.4
The max temperature difference (^{o}C)	69.6	34.6	14.3	2.4

Video S1: The graphene-PVDF bimorph actuator can swing quickly and reversibly with different frequencies (0.3, 0.6, 1.0 and 2.0 Hz) under the square wave voltage of 0-13.0 V. The dimension of the samples is 20 mm × 3 mm (length × width).

Video S2: A fishlike robot was designed to imitate fish swimming in petroleum ether, which was driven by the graphene-PVDF bimorph actuator. It can move under different frequencies (0.4, 0.8, and 2.0Hz). When the power (the square wave voltage of 0-13.0 V) is on or off, the "tail" will bend down or up, and then the fishlike robot will swim forward. The dimensions of the fish tail (the graphene-PVDF bimorph actuator) and the fish body (Expandable polystyrene) are 14 mm × 3 mm and about 30 mm × 8 mm (length × width), respectively.

[1] J. Liang, L. Huang, N. Li, Y. Huang, Y. Wu, S. Fang, J. Oh, M. Kozlov, Y. Ma, F. Li, R. Baughman, Y. Chen, *ACS Nano* **2012,** 6, 4508.

[2] Y. Han, Z. Xu, C. Gao, *Adv. Funct. Mater.* **2013,** 23, 3693.

[3] H. A. Becerril, J. Mao, Z. Liu, R. M. Stoltenberg, Z. Bao, Y. Chen, *ACS Nano* **2008,** 2, 463.

[4] M. Hirata, T. Gotou, S. Horiuchi, M. Fujiwara, M. Ohba, *Carbon* **2004,** 42, 2929.

[5] M.-m. Tao, F. Liu, B.-r. Ma, L.-x. Xue, *Desalination* **2013,** 316, 137.

[6] Y. K. A. Low, L. Y. Tan, L. P. Tan, F. Y. C. Boey, K. W. Ng, *J. Appl. Polym. Sci.* **2013,** 128, 2902.

[7] R. Gregorio, Jr., D. S. Borges, *Polymer* **2008,** 49, 4009.

[8] W. Ong, C. Ke, P. Lim, A. Kumar, K. Zeng, G. W. Ho, *Polymer* **2013,** 54, 5330.

[9] X. Xie, L. Qu, C. Zhou, Y. Li, J. Zhu, H. Bai, G. Shi, L. Dai, *ACS Nano* **2010,** 4, 6050.

[10] K. H. Im, H. Choi, *J. Korean Phys. Soc.* **2014,** 64, L623.
